# Immunogenetics of lithium response and psychiatric phenotypes in patients with bipolar disorder

**DOI:** 10.21203/rs.3.rs-3068352/v1

**Published:** 2023-06-26

**Authors:** Marisol Herrera-Rivero, Karina Gutiérrez-Fragoso, Anbupalam Thalamuthu, Azmeraw T. Amare, Mazda Adli, Kazufumi Akiyama, Nirmala Akula, Raffaella Ardau, Bárbara Arias, Jean-Michel Aubry, Lena Backlund, Frank Bellivier, Antonio Benabarre, Susanne Bengesser, Bhattacharjee Abesh, Joanna Biernacka, Armin Birner, Micah Cearns, Pablo Cervantes, Hsi-Chung Chen, Caterina Chillotti, Sven Cichon, Scott Clark, Francesc Colom, Cristiana Cruceanu, Piotr Czerski, Nina Dalkner, Franziska Degenhardt, Maria Del Zompo, J. Raymond DePaulo, Bruno Etain, Peter Falkai, Ewa Ferensztajn-Rochowiak, Andreas J. Forstner, Josef Frank, Louise Frisen, Mark Frye, Janice Fullerton, Carla Gallo, Sebastien Gard, Julie Garnham, Fernando Goes, Maria Grigoroiu-Serbanescu, Paul Grof, Ryota Hashimoto, Roland Hasler, Joanna Hauser, Urs Heilbronner, Stefan Herms, Per Hoffmann, Liping Hou, YiHsiang Hsu, Stéphane Jamain, Esther Jiménez, Jean-Pierre Kahn, Layla Kassem, Tadafumi Kato, John Kelsoe, Sarah Kittel-Schneider, Po-Hsiu kuo, Joachim Kurtz, Ichiro Kusumi, Barbara König, Gonzalo Laje, Mikael Landén, Catharina Lavebratt, Marion Leboyer, Susan Leckband, Mario Maj, Mirko Manchia, Cynthia Marie-Claire, Lina Martinsson, Michael McCarthy, Susan L. McElroy, Vincent Millischer, Marina Mitjans, Francis Mondimore, Palmiero Monteleone, Caroline Nievergelt, Tomas Novak, Markus Nöthen, claire odonovan, Norio Ozaki, Sergi Papiol, Andrea Pfennig, Claudia Pisanu, James Potash, Andreas Reif, Eva Reininghaus, Hélène Richard-Lepouriel, Gloria Roberts, Guy Rouleau, Janusz K. Rybakowski, Martin Schalling, Peter Schofield, Klaus Oliver Schubert, Eva Schulte, BARBARA SCHWEIZER, Giovanni Severino, Tatyana Shekhtman, Paul Shilling, Kazutaka Shimoda, Christian Simhandl, claire slaney, Alessio Squassina, Thomas Stamm, Pavla Stopkova, Fabian Streit, Fasil Ayele, Alfonso Tortorella, Gustavo Turecki, Julia Veeh, Eduard Vieta, Biju Viswanath, Stephanie Witt, Peter Zandi, Martin Alda, Michael Bauer, Francis McMahon, Philip Mitchell, Marcella Rietschel, Thomas Schulze, Bernhard Baune

**Affiliations:** University of Münster; University of New South Wales; University of Adelaide, AUSTRALIA; Department of Biological Psychiatry and Neuroscience, Dokkyo Medical University; National Institutes of Health, US Dept of Health & Human Services; Hospital University Agency of Cagliari; Facultat de Biologia and Institut de Biomedicina (IBUB), Universitat de Barcelona, CIBERSAM; Geneva University Hospitals; Mayo Clinic; McGill University Health Centre; National Taiwan University Hospital; Mayo Clinic; University of Adelaide; Max Plank Institute for Psychiatry; Poznan University of Medical Sciences; University of Bonn; University of Cagliari; Johns Hopkins University; University Hospital LMU; University of Bonn, School of Medicine & University Hospital Bonn; Central Institute for Mental Health; School of Medicine & University Hospital Bonn; Mayo Clinic; Neuroscience Research Australia; Alexandru Obregia Clinical Psychiatric Hospital; National Center of Neurology and Psychiatry; Institute of Psychiatric Phenomics and Genomics, University Hospital, LMU Munich; Institute of Human Genetics; National Institute of Mental Health Intramural Research Program, National Institutes of Health; Mayo Clinic; Univ Paris Est Creteil, INSERM, IMRB; School of Medicine & University Hospital Bonn; Laboratory for Molecular Dynamics of Mental Disorders, RIKEN Brain Science Institute, Wako, Saitama 351-0198, Japan; University of California, San Diego; College of Public Health, National Taiwan University, Taipei, Taiwan; Hokkaido University Graduate School of Medicine; Gothenburg University; Karolinska Institutet; University of Naples, Italy; Dalhousie University; INSERM UMR-S 1144; Karolinska Institutet; Lindner Center of Hope / University of Cincinnati; Max Planck Institute of Experimental Medicine, Göttingen, Germany; Neuroscience Research Australia; University of Salerno, University of Naples SUN; University of California, San Diego; National Institute of Mental Health, Klecany; School of Medicine & University Hospital Bonn; Nagoya University; University Hospital LMU; University Hospital Carl Gustav Carus, TU Dresden; University of Cagliari; University Hospital Frankfurt, Germany; McGill University; Poznan University of Medical Sciences; Karolinska Institutet; Neuroscience Research Australia; University of Adelaide; University Hospital, LMU Munich; University of Cagliari; Universita degli Studi Di Cagliari; Charité - Universitätsmedizin Berlin, Campus Charité Mitte; University of Heidelberg; Department of Psychiatry, University of Perugia, Perugia, Italy; Douglas Institute, Department of Psychiatry, McGill University; Hospital Clinic of Barcelona; National Institute of Mental Health and Neuro Sciences, Bengaluru, Karnataka, India.; University Medical Centre Mannheim; Johns Hopkins University; Dalhousie University; University Hospital Carl Gustav Carus; National Institute of Mental Health Intramural Research Program; National Institutes of Health; University of New South Wales; University of Mannheim; University of Munich; University of Münster

**Keywords:** bipolar disorder, immunity, lithium, polygenic scores, genetics

## Abstract

The link between bipolar disorder (BP) and immune dysfunction remains controversial. While epidemiological studies have long suggested an association, recent research has found only limited evidence of such a relationship. To clarify this, we investigated the contributions of immune-relevant genetic factors to the response to lithium (Li) treatment and the clinical presentation of BP. First, we assessed the association of a large collection of immune-related genes (4,925) with Li response, defined by the Retrospective Assessment of the Lithium Response Phenotype Scale (Alda scale), and clinical characteristics in patients with BP from the International Consortium on Lithium Genetics (ConLi^+^Gen, N = 2,374). Second, we calculated here previously published polygenic scores (PGSs) for immune-related traits and evaluated their associations with Li response and clinical features. We found several genes associated with Li response at p < 1×10^− 4^ values, including *HAS3, CNTNAP5* and *NFIB*. Network and functional enrichment analyses uncovered an overrepresentation of pathways involved in cell adhesion and intercellular communication, which appear to converge on the well-known Li-induced inhibition of GSK-3β. We also found various genes associated with BP’s age-at-onset, number of mood episodes, and presence of psychosis, substance abuse and/or suicidal ideation at the exploratory threshold. These included *RTN4, XKR4, NRXN1, NRG1/3* and *GRK5*. Additionally, PGS analyses suggested serum FAS, ECP, TRANCE and cytokine ligands, amongst others, might represent potential circulating biomarkers of Li response and clinical presentation. Taken together, our results support the notion of a relatively weak association between immunity and clinically relevant features of BP at the genetic level.

## INTRODUCTION

Bipolar disorder (BP) has been associated with some degree of immune dysfunction. Epidemiological data has linked immune-related medical comorbidities, including autoimmune and metabolic diseases, and chronic low-grade inflammation with BP. In particular, increases in pro-inflammatory cytokines are observed during affective episodes in patients with BP [1]. In addition, genomic studies have revealed weak yet significant genetic correlation between BP and immune-related diseases [2]. Nevertheless, as a number of these observations originated from underpowered studies [3], further investigations are required to elucidate the proposed relationships.

Lithium (Li), mainly used in the treatment of BP, is an effective pharmacological agent in the treatment of an array of psychiatric conditions [4, 5]. In addition to its mood-stabilizing effects, Li shows anti-viral and immune cell regulatory properties [6, 7]. The immune regulatory activity of Li has been partially attributed to the modulation of pro-inflammatory cytokines and GSK-3β. Therefore, it has been suggested that the mechanism through which Li improves symptom progression may be via anti-inflammatory effects [8, 9]. The Retrospective Assessment of the Lithium Response Phenotype Scale (Alda scale) is the most widely used clinical measure of Li response. Most often, it is dichotomized such that individuals with scores ≥ 7 are classified as “responders” and those with scores < 7 as “non-responders” [10, 11]. Using this metric, previous genetic studies have implicated human leukocyte antigen (HLA) and inflammatory cytokine genes in the response to Li treatment in BP [12, 13]. Therefore, we hypothesized that single nucleotide polymorphisms (SNPs) in immune-related genes contribute, to some extent, to Li response and further, may impact specific clinical features within BP. To test our hypothesis, we performed association studies of a comprehensive collection of immune-related genes in 2,374 patients with BP from the International Consortium on Lithium Genetics (ConLi^+^Gen) [14]. Additionally, we tested associations with published polygenic scores (PGSs) for immune-relevant traits.

## METHODS

Since our study follows a candidate approach to selected genes, pathways and networks, a diagram summarizing the methodology employed can be found in the Supplementary Figures: Figure S1.

### Study sample

The ConLi^+^Gen cohort has been previously described in detail [15]. Briefly, peripheral blood samples from individuals with a diagnosis of a bipolar spectrum disorder (in accordance with the criteria established in the Diagnostic and Statistical Manual of Mental Disorders -DSM- versions III or IV) that had taken Li for a minimum of six months (with no additional mood stabilizers), were collected from 2003 to 2013. The isolated DNA was genotyped in two phases. This resulted in two sample batches originally referred to as “GWAS1” and “GWAS2”, comprising 1,162 and 1,401 individuals, respectively. Long-term responses to Li treatment were assessed in both sample batches using the Alda scale. Here, the A subscale rates the degree of response on a 10-point scale, and the B subscale reflects the relationship between improvement and treatment. A total score, ranging from 0–10, is obtained by subtracting the B score from the A score of these subscales. Negative scores are set to 0. Data on age-at-onset (AAO), age (at sample collection and phenotyping), sex and diagnostic subtype were available for both sample batches. Diagnoses included bipolar disorder type I and type II, schizoaffective bipolar disorder and bipolar disorder not otherwise specified. Additionally, information on psychiatric features, namely the number of episodes of depression, mania and hypomania, the presence of psychosis, alcohol and substance abuse, and suicidal ideation, were available for patients in the “GWAS1” batch.

The Ethics Committee at the University of Heidelberg provided central approval for the ConLi^+^Gen Consortium. Written informed consent from all participants was obtained according to the study protocols of each of the participating sites and their institutions. All procedures were performed in accordance with the guidelines of the Declaration of Helsinki.

### Immune gene collection

A comprehensive set of immune-related genes was collated from gene lists available in the online databases MSigDB [16] and InnateDB [17]. From MSigDB (https://www.gsea-msigdb.org/gsea/msigdb/), the following gene sets contained in the C2 “curated gene sets” collection were retrieved: M1036: Reactome-innate immune system, M1058: Reactome-adaptive immune system, M39895: WikiPathways (WP)-neuroinflammation, M39711: WP-cytokines and inflammatory response, and M39641: WP-inflammatory response pathway. From InnateDB (https://www.innatedb.com/index.jsp), the curated gene lists derived from the Immunology Database and Analysis Portal (ImmPort), the Immunogenetic Related Information Source (IRIS) and the Immunome Database, were downloaded. Chromosomal locations were annotated from Ensembl using the hg19 build. Herein, the combined collection is referred to as the ImmuneSet and contained 4,925 autosomal genes to be included in association analyses.

### Genotype data

Schubert et al. (2021) [18] have previously described the creation of the genotype dataset used herein. Briefly, DNA samples were originally genotyped using either Affymetrix or Illumina SNP arrays. These genotype data from multiple cohorts were separately imputed using the 1000 Genomes Project reference panel phase 3 v5. Each imputed dataset underwent a basic quality control (QC) step to keep variants with minor allele frequency (MAF) > 0.01, Hardy-Weinberg equilibrium p-value (HWE) ≥ 1×10^− 6^ and imputation quality score (Rsq) ≥ 0.6. Genotype calls were derived from the imputed dosage scores and all datasets were merged by retaining only common sets of SNPs. To update this dataset and obtain a higher number of good quality variants, we re-imputed the genotype data via the Michigan Imputation Server [19] using the Haplotype Reference Consortium (HRC) panel for European ancestry. The re-imputed genotypes underwent a QC step to keep variants with Rsq ≥ 0.8, MAF ≥ 0.01 and HWE ≥ 1×10^− 6^. Additionally, individuals were removed if they failed the heterozygosity test and/or showed relatedness, according to the tests performed using the plinkQC R package [20]. In the latter case, one individual from each pair of related individuals (PI-HAT > 0.25) was removed. For analysis of the ImmuneSet, SNPs within each gene’s boundaries (± 0 kb) were retained. The final ImmuneSet genotype datasets contained 701,031 SNPs from 1,024 and 1,350 individuals in “GWAS1” and “GWAS2”, respectively.

### Polygenic Scores

A set of 32 published PGSs available at the PGS Catalog [21] were used to approximate markers of inflammation and immune-related phenotypes that were not experimentally measured in the “GWAS1” ConLi^+^Gen sample. These PGSs, created and evaluated in large samples of predominantly European ancestry, stemmed from three recent publications and corresponded to the following traits: autoimmune disease [22], lymphocyte / monocyte / eosinophil / neutrophil / basophil percentage of white (blood) cells [23], and serum levels of 26 markers of inflammation [24]. After downloading and harmonizing weight files, we performed allelic scoring in ConLi^+^Gen using the sum method applied in Plink 1.9 [25].

### Association analyses

The “GWAS1” (N = 853) and “GWAS2” (N = 1,258) samples were tested separately for associations of 701,031 SNPs in the ImmuneSet with: 1) Li response (responder / non-responder, defined by Alda scores ≥ 7 or < 7, respectively), 2) total Alda score, 3) Alda subscale A, and 4) Alda subscale B (total). Because the most reliable continuous Li response phenotype has been previously shown to be the Alda A score, when excluding individuals with Alda B scores > 4 [10], we tested this as the primary continuous phenotype in our study. All association tests were performed applying an additive model in Plink 1.9, and all models were adjusted for age at recruitment, age-at-onset (AAO), sex, diagnosis and the first principal components (PCs) obtained for each ImmuneSet genotypes dataset. PCA plots were explored to determine the optimal number of PCs to be used as covariates for each sample. Therefore, the first five PCs were used as covariates for “GWAS1” while the first six PCs were used for “GWAS2”. Population stratification due to ancestry was successfully corrected by the selected numbers of PCs (Supplementary Figures: Figure S2). Next, the association results for Li response from “GWAS1” and “GWAS2” samples were meta-analyzed using the weighted-z (METAL) method applied in Plink 1.9. These meta-analysis results were QCed to exclude variants with I^2^ heterogeneity index (I) > 40 and p-value for Cochran’s Q statistic (Q) < 0.1 (highly heterogeneous). We searched first for associations at the commonly accepted thresholds for GWASs (genome-wide significance, p < 5×10^− 8^, and suggestive significance, p < 1×10^− 5^). However, considering this a candidate gene rather than a genome-wide approach, we chose to look further into findings with p < 1×10^− 4^, a threshold that has been previously used to select association findings for follow-up in GWASs [26] and, therefore, represents an acceptable exploratory threshold.

In “GWAS1”, the ImmuneSet was further tested for associations with other BP clinical phenotypes (i.e. AAO, the number of episodes of depression, mania and hypomania, as well as the presence of psychosis, alcohol and/or substance abuse, and suicidal ideation). These models were adjusted for age at recruitment, AAO (except when AAO was tested as phenotype), sex, diagnosis and the first five PCs. Statistical significance was considered as above.

Associations between PGSs and the various BP clinical phenotypes were tested using linear or binomial regression models, as appropriate, adjusted for age at recruitment, AAO (except when AAO was tested as phenotype), sex, diagnosis and population using the robustbase R package. Significance was set to false discovery rate (FDR) < 0.05. However, we also looked into the nominally significant findings (p < 0.05) for exploratory purposes.

### Downstream analyses

All variants under the p < 1×10^− 4^ threshold were annotated for known regulatory effects on gene expression (i.e. expression quantitative trait loci, eQTLs) in all human brain, blood, spleen and thyroid tissues, as well as in immune cells (e.g. monocytes and macrophages) using Qtlizer [27].

A protein-protein interaction (PPI) network to explore the functional relevance of the identified genes associated with Li response was created using the ReactomeFIViz app [28] for Cytoscape 3.7 [29]. This analysis used as input a list composed of the ImmuneSet genes showing associations at the p < 1×10^− 4^ threshold with the dichotomous and continuous Li response phenotypes. The network also incorporated “linker” genes (i.e. genes not in the input gene list that create indirect connections between input genes) to increase biological interpretability. Moreover, pathway overrepresentation analysis was performed on the PPI network (including linker genes) using the pathway enrichment network function of the app. Because the linker genes were not drawn from the ImmuneSet collection, we used the standard background genes of the ReactomeFiViz app for this analysis. The resulting overrepresented pathways were filtered to exclude terms that: 1) had FDR > 0.05, 2) corresponded to a specific disease (e.g. bladder cancer, herpes virus infection), 3) had less than two genes overlapping between the pathway set and the network set, and/or 4) the overlap with the pathway set represented less than 3% of genes in the set. Additionally, we repeated the pathway overrepresentation analysis including not only the variant mapped genes, but also the annotated eQTL genes.

For associations with clinical phenotypes in the “GWAS1” sample, functional analyses were performed using the GENE2FUNC tool of the Functional Mapping and Annotation of Genome-Wide Association Studies (FUMA-GWAS) platform [30]. The input gene lists included mapped and eQTL genes annotated for variants below the p < 1×10^− 4^ threshold for each studied phenotype. Because eQTL genes were not drawn from our ImmuneSet collection, we used all protein-coding genes as background for these analyses. Overrepresented gene sets were those that showed FDR < 0.05, following a hypergeometric test, and a minimum of two overlapping genes. Curated gene sets from pathway databases in the “canonical pathways” category were preferred when available. Otherwise, Gene Ontology biological processes (GO_BPs) or any other available category (including GWAS Catalog trait associations) were taken. For GTEx-based enriched tissues of expression, as our focus is on immune-brain relationships, we kept only those enrichments corresponding to brain expression, as these are the most relevant tissues for the analysis of Li response and clinical features of BP.

In addition, the relative importance for (dichotomous) Li response of the calculated PGSs in “GWAS1” was assessed through a machine learning (ML) screening approach using the Auto Model extension of RapidMiner Studio. This applied various classification algorithms to the raw PGS data. Auto Model provides the following models: Naïve Bayes, Generalized Linear Model, Logistic Regression, Fast Large Margin, Deep Learning, Decision Tree, Random Forest, Gradient Boosted Trees and Support Vector Machines. Because ML algorithms are sensitive to class imbalance, an equal number of responder and non-responder individuals were randomly selected for the analysis (N = 657) using the sample method of the Python’s Pandas library. The resulting file with balanced classes was used as input for the ML screening in Auto Model, where the sample was randomly divided into training (60%) and test (40%) sets. Default parameters for all algorithms were applied. Given that different types of ML algorithms can differ in their feature selection procedure due to their inherent characteristics, here, features were considered important for Li response, with either a positive (i.e. favoring response) or a negative (i.e. favoring non-response) effect, when at least two algorithms selected the same feature with the same effect direction as important for the classification task.

## RESULTS

### Immune-related genes associated with response to Li treatment in BP

After excluding individuals with missing phenotypic data (age and/or AAO), the effective sample sizes for the association analyses in ConLi^+^Gen were 853 and 1,258 in “GWAS1” and “GWAS2”, respectively. A basic description of both samples is shown in Table 1. In general, there were more female than male patients in both samples and there were minimal differences in the mean ages at recruitment and disease onset between “GWAS1” and “GWAS2”. Therefore, the total sample size of our meta-analyses of Li response was 2,111, including 606 (28.7%) responders and 1,505 non-responders for the dichotomized variable, which included 1,224 (58%) females and 887 males, with mean age 47 (± 14) years and mean AAO 25 (± 11) years. For the continuous Li response phenotype (i.e. Alda A score, excluding individuals with Alda B score > 4), the effective sample sizes were 828 for “GWAS1” and 1,044 for “GWAS2”. After the post-meta-analysis exclusion of variants with heterogeneous effects between both ConLi^+^Gen samples, a mean of 556,196 SNPs remained in each set of summary statistics. This was higher for the continuous phenotype, in which 625,818 SNPs remained.

We found no associations with Li response at the genome-wide GWAS threshold (p < 5×10^− 8^). At the suggestive threshold for GWAS (p < 1×10^− 5^), the dichotomous Li response phenotype and Alda B (total) showed associations with *FAT3* (best SNP: rs4313539, p = 2.1×10^− 6^, z = 4.744), and with *ADAMTS5* (best SNP: rs162501, p = 9.18×10^− 7^, z = 4.909) and *GRID2* (best SNP: rs62312225, p = 2.71×10^− 6^, z = 4.692), respectively (Supplementary File 1: Table 1). At the exploratory threshold (p < 1×10^− 4^), when considering linkage disequilibrium (LD), we identified between 9 and 12 genomic loci in relation with different aspects of Li response (Supplementary File 1: Table 1). The most significant SNPs from the analyses of the dichotomous and continuous phenotypes mapped to *FAT3* and *BMPR1A*, respectively (Table 2). In total, 42 genes were implicated in the response to Li in patients with BP from our exploratory analyses in the ConLi^+^Gen cohort (Supplementary File 2: Table 1). As expected, there was a number of gene-based overlaps between different aspects of Li response, particularly between the dichotomous variable and total Alda score, and between the continuous variable and the other Alda variables.

**Figure 1:**
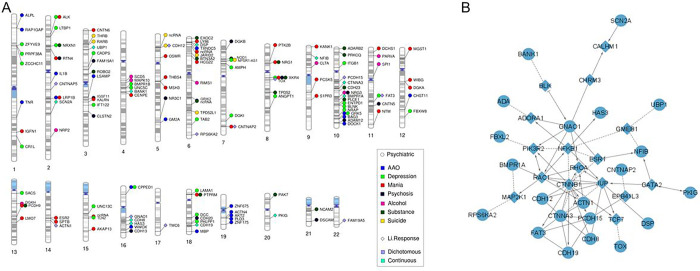
Exploratory findings for the ImmuneSet in ConLi^+^Gen. **A)** Gene-based phenogram of associations of the ImmuneSet with Li response and clinical features in ConLi^+^Gen. **B)** Protein-protein interaction network of Li response phenotype associations in the ImmuneSet. Circles represent input genes and diamonds represent linker genes. Dotted lines denote predicted interactions.

**Figure 2:**
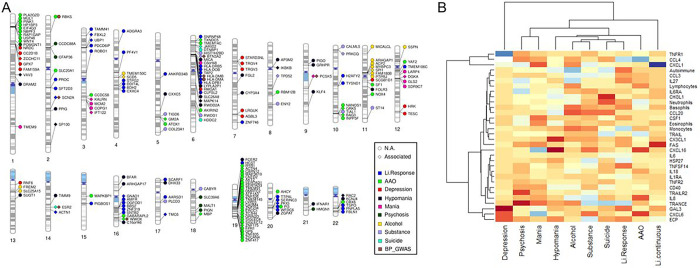
**A)** Gene-based phenogram of eQTL annotations for the exploratory-level findings of the ImmuneSet in ConLi^+^Gen. Those eQTL genes that were different from the mapped gene and those that were the same are presented in circle and diamond shapes, respectively. Only protein-coding genes are shown. In addition, overlaps with BP genetic associations reported in the GWAS Catalog are presented. **B)** Exploratory findings for the immune-related polygenic scores calculated in ConLi^+^Gen. Significance of calculated PGSs for BP phenotypes in ConLi^+^Gen “GWAS1”. The heatmap shows the z-values obtained for each PGS-phenotype pair. Increasing color darkness alludes to increasing effect, with red and blue colors at the negativ and positive extremes, respectively.

Twenty-four ImmuneSet genes were implicated in the primary Li response phenotypes (i.e. dichotomous and continuous) in our exploratory analysis (Fig. 1A). These were used as input for a network analysis to facilitate biological interpretation of the findings. This network analysis provided known and predicted functional interactions between a subset of 21 input genes from our association results and 16 linkers drawn from the total of protein-coding genes in the background reference of the ReactomeFiViz app (Fig. 1B). Functional analysis of the network uncovered an overrepresentation of crucial developmental pathways and regulatory networks, suggesting the involvement of processes such as assembly and stability of the cell-cell signaling machinery (e.g. adherens junction, E-cadherin signaling, focal adhesion, integrin signaling, L1 cell adhesion molecule signaling), neuronal development and function (e.g. neurotrophic signaling, lysophosphatidic acid receptor mediated events, regulation of pluripotency, Wnt signaling), as well as activation of inflammatory (e.g. sphingolipid signaling, S1P pathways, toll-like receptor signaling) and adaptive immune pathways (e.g. T and B cell receptor signaling) in the biological response to Li. Interestingly, processes such as angiogenesis, long-term potentiation, thyroid hormone signaling pathways, melanogenesis and sensory processing were also overrepresented in our network analysis (Supplementary File 2: Table 2). These observations suggest that variation in immune-related genes and its effects that expand beyond inflammatory and immune responses from early developmental stages contribute to determine the extent of the organism’s response to Li treatment in patients with BP. Moreover, when eQTL genes were incorporated into the analysis, there was a marked overrepresentation of inflammatory and autoimmune disease pathways (e.g. asthma, type 1 diabetes mellitus, autoimmune thyroid disease, inflammatory bowel disease) and of vitamin D metabolism. The Wnt signaling, cell adhesion and adaptive immune pathways remained significantly overrepresented (data not shown). All protein-coding eQTL genes annotated for Li response phenotypes are shown in Fig. 2A.

### Immune-related genes associated with clinical phenotypes in BP

The association analyses of the ImmuneSet with specific clinical features that were available for the “GWAS1” sample showed no associations at the genome-wide GWAS threshold. However, at the suggestive GWAS threshold there were, collectively, 100 associations between the ImmuneSet and AAO (3), number of depressive (54) and manic (14) episodes, and the presence of psychosis (1), substance use disorder (15) and/or suicidal ideation (13). These implicated 17 genes that associated to specific clinical features (i.e. no overlaps were observed at this threshold Table 3). When we moved forward to the exploratory analysis, we identified 786 SNP-phenotype associations in total (Supplementary File 1: Tables 2–9). These implicated 166 immune-related genes (Fig. 1A; Supplementary File 2: Table 1) mostly involved in adaptive immunity and inflammation. Beyond their immune functions, however, these genes play important roles in the development of the nervous system, signal transduction, synaptic processes and cell adhesion (Supplementary File 2: Tables 3–9). In particular, large numbers of associations were found for AAO and mood episodes (Table 3). In addition, 156 eQTL genes were collectively annotated for these phenotypes. Figure 2A shows the protein-coding eQTL genes annotated for each clinical feature. Even when these showed no overlaps among the clinical phenotypes, there were some overlaps with the ImmuneSet genes meaning that, in some instances, the eQTL gene corresponded to the gene mapped to the variant while, in others, the eQTL gene was different from the mapped gene. A summary of exploratory findings for each clinical phenotype studied is shown below.

#### Age-at-onset.

Fifty-four associations in 21 ImmuneSet genes were found for AAO in our study (Table 3, Supplementary File 1: Table 2). These genes were enriched for negative regulation of cell death and synaptic transmission, as well as expression in the cerebellum (Supplementary File 2: Table 3). The top variant, rs1248079 (p = 3.9×10^− 6^, beta = 2.75), mapped to an intronic region in *GRK5* (G Protein-Coupled Receptor Kinase 5). Other important genes included *PLD3, AKT2* and *IL1B*. The addition of eQTL genes to the functional enrichment analysis resulted in an additional overrepresentation of processes related to cellular stress responses.

#### Depression.

With an effective sample of 692 individuals, 107 associations in 31 ImmuneSet genes for the number of depressive episodes were found (Table 3, Supplementary File 1: Table 3). These genes were enriched for synaptic processes, as well as expression in frontal and anterior cingulate cortices (Supplementary File 2: Table 4). While the top variant, rs55975329 (p = 1.7×10^− 7^, beta = 4.54), localized to an intron in *BLNK* (B cell linker), other important genes included *PHLPP1, ZCCHC11 (TUT4)* and *CPPED1*. The addition of eQTL genes to the functional enrichment analysis resulted in an additional overrepresentation of axonal and synaptic components.

#### Hypomania.

Although the largest associations were found for the number of hypomanic episodes, these observations were based on only 85 individuals with available data. Therefore, we have excluded this phenotype from the figures and tables shown within this manuscript. All corresponding results are provided in the supplementary material (Supplementary File 1: Table 4, Supplementary File 2: Table 5).

#### Mania.

With an effective sample of 665 individuals, 116 associations in 32 ImmuneSet genes for the number of manic episodes were found (Table 3, Supplementary File 1: Table 5). These genes were enriched for neuronal development and differentiation, as well as expression in spinal cord and frontal cortex (Supplementary File 2: Table 6). The top variant, rs59134172 (p = 2.4×10^− 6^, beta = 1.62), localized to an intron in *CNTN6* (Contactin 6). Other important genes included *KALRN, PCDH9, PTK2B* and *CNTNAP2*. Interestingly, we also found enrichment for response to serotonin re-uptake inhibitors in major depressive disorder (FDR = 0.042) and serum thyroid-stimulating hormone levels (FDR = 0.0028), from the GWAS Catalog trait associations, in mania-associated ImmuneSet genes. The addition of eQTL genes to the functional enrichment analysis had no impact on the overrepresented gene set classes.

#### Psychosis.

The effective sample for our analysis of the presence of psychosis in BP was 692 individuals. Here, 45 associations in 13 genes were identified (Table 3, Supplementary File 1: Table 6). The implicated genes were enriched for cell adhesion and synapse organization, as well as expression in frontal cortex (Supplementary File 2: Table 7). The top SNP, rs459374 (p = 7.6×10^− 6^, beta = 1.96), is located in an intronic region of *DSCAM* (DS Cell Adhesion Molecule). Other interesting genes included *CLSTN2, RTN4* and *CDH13.* Moreover, the GWAS Catalog traits seasonality and depression (FDR = 0.02), and response to amphetamines (FDR = 0.026), as well as obesity-related traits (FDR = 0.015), atrial fibrillation (FDR = 0.027) and diastolic blood pressure (FDR = 0.027) were enriched among the psychosis-associated genes. The addition of eQTL genes to the functional enrichment analysis had no impact on the overrepresented processes. However, this resulted in a considerable increase in overepresented brain tissues of expression, including the hippocampus, amygdala, hipothalamus, anterior cingulate cortex, putamen and substantia nigra.

#### Alcohol and substance abuse.

The effective sample sizes for alcohol and substance use disorders in ConLi^+^Gen were 835 and 832, respectively. Twenty-nine SNPs in nine genes were associated with alcohol use, with the rs7698751 SNP in *SCD5* (Stearoyl-CoA Desaturase 5) being the top association (p = 1.8×10^−5^, beta = 2.61). Although no gene set enrichments were found for associations with alcohol abuse, other implicated genes included the BP-associated *DGKH, NRG3* and *RIMS1* (Supplementary File 1: Table 7). Interestingly, when incorporating the eQTLs genes into this functional analysis, overrepresentation of genes associated with mood swings, loneliness and anxious behaviors in the GWAS Catalog was observed (Supplementary File 2: Table 8). For substance use disorder, 78 associations implicating 17 genes were found (Supplementary File 1: Table 8). Genes were overrepresented in neurogenesis-related processes, with expression in cerebellum as well as frontal and anterior cingulate cortices (Supplementary File 2: Table 8). The top variant, rs7814474 (p = 4.5×10^− 7^, beta = 6.7), was mapped to an intron in *TPD52* (Tumor Protein D52). Other genes included the BP-associated *NRG1*, the schizophrenia-associated *PTPRM*, as well as *NOD1* and *PRKCQ*. In addition, the GWAS Catalog traits chronotype (FDR = 0.011) and serum thyroid-stimulating hormone levels (FDR = 0.047) were also overrepresented in substance abuse-associated ImmuneSet genes. The addition of eQTL genes to the functional enrichment analysis resulted in an additional overrepresentation of the phosphatidylinositol signaling system and expression in the amygdala, hippocampus and basal ganglia.

#### Suicidal ideation.

Information on the presence of suicidal thoughts was available for 660 ConLi^+^Gen individuals. Based on these, 30 variants in seven genes were associated with suicidal ideation in the “GWAS1” sample (Table 3, Supplementary File 1: Table 9). The top SNP was rs2327882 (p = 3.9×10^− 6^, beta = 0.55), located in an intron of the *JARID2* (Jumonji and AT-Rich Interaction Domain Containing 2) gene. Gene set enrichment analysis found overrepresentation of functions of nuclear receptors (Supplementary File 2: Table 9). Indeed, these terms related to *RARB* and *THRB*. The addition of eQTL genes to the functional enrichment analysis had no impact on the overrepresented gene set classes.

### Immune-related genes showed pleiotropy for BP phenotypes

Not surprisingly, a number of genes showed shared associations with the different phenotypes included in our exploratory ImmuneSet analyses in ConLi^+^Gen (Fig. 1A). Therefore, these genes can be prioritized for follow-up studies due to their pleiotropic effects in Li response, clinical features, or both. In this way, we prioritized 21 genes that showed associations with more than one BP phenotype (Table 4). Here, we excluded genes associated with Alda A, total Alda B and/or Total Alda when there was no overlap with the dichotomous and/or continuous Li response phenotypes. However, a complete list of corresponding gene-phenotype associations (197 in total) is shown in Supplementary File 2: Table 1. Five of the prioritized genes (*CNTNAP5, DSP, NFIB, BMPR1A*, and *HAS3*) were associated with multiple Li response phenotypes, 10 of them (*XKR4, NRXN1, RTN4, NRG1/3, ALK, GRK5, LRP1B, NPSR1-AS1* and *CPPED1*) were associated with multiple clinical features, and another six genes (*BANK1, ROBO2, CNTNAP2, PCDH9, CDH12* and *FAT3*) were associated with Li response as well as with clinical features.

### Polygenic scores for immune-related traits associated with BP phenotypes

In addition to testing associations of the ImmuneSet with BP phenotypes, we calculated a set of 32 previously published immune-related PGSs, namely for: 1) (general) autoimmune disease, 2) the proportions of white blood cell populations and 3) inflammatory marker levels in serum (Supplementary File 2: Table 10). The overlap of variants between the PGS weight files obtained from PGS Catalog and the SNPs available in our ConLi^+^Gen “GWAS1” sample was, in general, better for the serum levels of inflammatory markers (80.6% in average) than for the other PGSs. The lowest valid SNP overlap was found for the PGS of general autoimmune disease (38.8%), suggesting that this PGS may relatively poorly index autoimmune disease signatures. For the proportion of white blood cell populations, the valid SNP overlap was also not fully satisfactory (49.4% in average). Nevertheless, we were able to obtain some valuable insights by using these scores (Fig. 2B). We found associations (FDR < 0.05) of the PGSs for CXCL1 (C-X-C Motif Chemokine Ligand 1; z = 14.9, p < 2×10^− 6^), ECP (Eosinophilic Cationic Protein; z = 3.7, p = 2.4×10^− 4^), FAS (Fas Cell Surface Death Receptor; z= −5.9, p = 5.6×10^− 9^) and TRANCE/TNFSF11 (TNF-related Activation-induced Cytokine/TNF Superfamily Member 11; z = 3.4, p = 0.0007) with the continuous Li response variable. No associations with the dichotomous variable survived correction for multiple comparisons. However, ML-based PGS ranking for the dichotomized Li response added another level of support to the links with CCL4 and ECP, and suggested relative importance of HSP27 (Heat Shock Protein Family B (Small) Member 1), CHI3L1 (Chitinase 3 Like 1), TNFR1, TRAILR2 and TNFSF14 (TNF Superfamily Member 14) for the prediction of responses to Li treatment in ConLi^+^Gen (Supplementary Figures: Figure S3). The PGSs for CCL4 (C-C Motif Chemokine Ligand 4; z = 5.4, p = 7.5×10^− 8^) and TRAIL/TNFSF10 (TNF-related apoptosis-inducing ligand/TNF Superfamily Member 10; z = 3.6, p = 3.7×10^− 4^) were associated with disease AAO. For the number of episodes of depression, we found associations with the PGSs for CXCL1 (z = 6.5, p = 1.3×10^− 10^), CXCL6 (C-X-C Motif Chemokine Ligand 6; z= −12.2, p < 2×10^− 6^), ECP (z= −11.8, p < 2×10^− 6^), GAL3 (Galectin 3; z= −20.1, p < 2×10^− 6^), and TNFR1/TNFRSF1A (TNF Receptor Superfamily Member 1A; z = 21.9, p < 2×10^− 6^). The number of episodes of mania were associated with the PGSs for eosinophils (z= −2.9, p = 0.003), CSF1 (Colony Stimulating Factor 1; z= −3.4, p = 7.7×10^− 4^), CXCL1 (z= −4.1, p = 4.6×10^− 5^), CXCL6 (z = 3.9, p = 1.1×10^− 4^), CXCL16 (z = 3.9, p = 1.2×10^− 4^), FAS (z= −3.3, p = 0.0012) and TRANCE (z= −3.8, p = 1.5×10^− 4^). Finally, the PGSs for TRAILR2/TNFRSF10B (TNF Receptor Superfamily Member 10b; z= −4.6, p = 5.1×10^− 6^) and TRANCE (z= −5.2, p = 2.1×10^− 7^) were associated with the presence of psychosis in patients with BP. A number of associations between PGSs for immune-relevant blood traits and clinically-relevant BP features remained only suggested, as these did not survive correction for multiple comparisons (Supplementary File 2: Table 10).

## DISCUSSION

There is apparent mounting evidence of immune dysregulation in BP and other major psychiatric diseases. Nevertheless, some observations have originated from underpowered studies, resulting in a lack of reproducibility [3]. Therefore, it becomes crucial to gain a better understanding of the relationships between the immune and central nervous systems, and to discern between causes and consequences of disease. In very general terms, it can be assumed that the existence of genetic associations with a given phenotype indicates causal contributions of the associated loci to the studied phenotype. With this in mind, we sought to investigate how genetic factors relevant to immune activity relate to disease phenotypes, such as response to Li treatment, AAO and psychiatric symptoms, in patients with BP. Using an exploratory and extensive candidate gene approach, our study prioritized various genes and inflammatory markers that appeared to represent pleiotropic factors contributing to multiple phenotypes. However, we should note that, here, we refer to pleiotropy as the (suggested) association with multiple traits in the ConLi^+^Gen cohort and, by no means, have we implied that these features are independent from each other. In fact, it should be expected that, given the important correlation between psychiatric disorders, the features that we have studied in ConLi^+^Gen are, to some extent, also correlated with each other. Also, because genes can play different roles in different tissues and cell types, we observed widespread enrichments of biological pathways participating in the development and function of the brain. This suggested that the genes implicated in BP phenotypes might affect in parallel both the immune and nervous systems. Nevertheless, because we found no associations at the genome-wide GWAS threshold of significance, and those observed at the suggestive GWAS threshold were limited, our findings are also consistent with a relatively weak effect of immune genetic factors over BP phenotypes.

The results of our exploratory assessment of associations of genetic polymorphisms in immune-related genes with Li response in the ConLi^+^Gen cohort suggest that variations in inflammatory and adaptive immune processes might contribute to the efficiency of the response to Li treatment in patients with BP. Importantly, our network and gene set enrichment analysis uncovered an involvement of numerous biological pathways that participate in cell adhesion, migration and intercellular communication, helping in the development and maintenance of the central and peripheral nervous systems, as well as of the immune and vascular systems. Interestingly, many of these appear to converge in the participation of GSK-3β (glycogen synthase kinase-3 beta), as assessed through comparative overlap analysis of the enriched KEGG and Reactome gene sets. GSK-3β is involved in multiple major developmental pathways, such as the Wnt, Notch and Hedgehog signaling pathways. Genetic manipulation in mouse models has shown an antidepressant-like behavior upon GSK-3β knockdown in hippocampus, as well as cognitive, behavioral and biochemical changes associated with psychiatric disorders, including Alzheimer’s disease, BP and schizophrenia, upon GSK-3β overexpression [31]. Li possesses a well-known inhibitory effect over GSK-3β [32]. Therefore, our analysis suggests that GSK-3β might be highly relevant for the response to Li treatment in BP. Our findings are also in agreement with other epidemiological and molecular investigations of Li effects. For example, we repeatedly observed overrepresentation of gene sets related to thyroid function, such as thyroid-stimulating hormone signaling and autoimmune thyroid disease. This is in line with the reports of a reversible association of Li treatment with hypothyroidism, particularly in women [33, 34].

Taken together, the encouraging literature supporting our findings sparked our interest in exploring how a genetic measure (PGS) for inflammatory marker levels in serum might associate with Li response in ConLi^+^Gen. Our results supported a relationship between Li treatment response and the genetic influence on the levels of various inflammatory markers circulating in serum. These included CXCL1, ECP, FAS, TRANCE and CCL4, molecules that regulate the activation and recruitment of immune cells (T and B lymphocytes, monocytes, macrophages, neutrophils and eosinophils).

The results of our exploratory assessment of associations of genetic polymorphisms in the ImmuneSet genes with BP’s AAO, numbers of mood episodes and psychiatric comorbidities in ConLi^+^Gen identified various candidate genes particularly contributing to mood episodes and substance abuse, including *XKR4, NRXN1*, *GRK*5 and *NRG1/3*. These genes, besides their immune-related functionalities, seem to play important roles in neuronal development and function, according to our gene set enrichment analyses. Indeed, this could be corroborated by the literature in many instances. For example, *NRXN1*, a cell surface protein involved in cell-cell interactions, exocytosis of secretory granules and regulation of signal transmission, has been associated with autism, schizophrenia and nicotine dependence [35]. *GRK5* has a role in the regulation of motility in polymorphonuclear leukocytes and inflammation [36, 37]. It also regulates the activity of various G-protein coupled receptors, including neurotransmitter receptors [38]. *XKR4*, a phospholipid scramblase strongly expressed in brain tissue and activated by caspases, has been suggested to participate in the remodeling of neural networks by triggering microglial responses to the exposure of phosphatidylserine on axons, dendrites and synapses [39].

It is worth noting that three of our candidate genes for depressive episodes have previously shown associations with either major depression at the gene-level (*DCC*) [40] or mapped to schizophrenia loci (*CR1L* and *DGKI*) [41] in large GWASs. Moreover, one of our candidate genes for manic episodes, *ESR2*, and one for substance use disorder, *BTN3A2*, were previously associated at the gene-level with major depression in the large GWAS, while one of our candidates for hypomanic episodes and alcohol dependence, the BP-associated *RIMS1* [26], mapped previously to schizophrenia loci. This, together with the overlaps with reported BP-associated genes that we observed, particularly concerning known eQTL genes, provides another level of support to the validity of our findings.

Additionally, the results of our exploratory assessment of PGS associations led to interesting observations. For example, that activation of macrophages and neutrophils, reflected by the associations with the PGSs for various cytokines and chemokines produced by or targeting these cells (e.g. interleukin-8, CX3CL1, CXCL6/16), might contribute to disease AAO in ConLi^+^Gen. Indeed, these observations are supported by studies that have found increases in neutrophil counts in psychiatric disorders, including BP [42, 43], as well as association of genetic polymorphisms in interleukin-1β, a pro-inflammatory cytokine produced by activated immune cells, including neutrophils and macrophages, with age of onset of depression in geriatric patients [44]. In addition, if we consider that macrophage/neutrophil activation was also a suggested mechanism of Li response in our study, it would be easy to speculate that activation of these cells might be linked with some aspect of the disease onset.

In conclusion, we performed an exploratory study that indicates a relationship between immunity and clinically relevant BP phenotypes at the genetic level, and pinpoints various interesting candidates for follow-up studies. We acknowledge that our study was limited by a relatively small sample size, particularly for the episodes of hypomania, and by incomplete overlap between the variants in the PGSs and our ConLi^+^Gen dataset. The latter, which likely resulted from a limited overlap among the different SNP arrays initially used to genotype samples in different collection centers, caused an incomplete indexing of the immune phenotypes of interest, and a relatively low rate of survival of correction for multiple comparisons in the PGS-BP phenotype relationships. Nevertheless, despite inherent limitations, we believe that our study provides valuable insight and furthers the understanding of the immune implications in BP. These results complement the evidence coming from epidemiological data and previous findings in ConLi^+^Gen, and support the hypothesis that, to some extent, immune regulation might represent a feasible strategy to improve the symptomatology and treatment response in patients with BP.

## Figures and Tables

**Table 1:** Description of the ConLi^+^Gen samples.

	Responders (Total Alda ≤ 7)	Non-Responders (Total Alda < 7)	Total
GWAS1			
N Effective sample (% from total)	297 (34.8)	556 (65.2)	853
N Females (%)	178 (59.9)	337 (60.6)	515 (60.4)
Age (mean ± SD)	52 ± 14	46 ± 14	48 ± 14
Age-at-onset (mean ± SD)	28 ± 11	23 ± 11	25 ± 11
# Depressive episodes (mean)	5	7	6
# Hypomanic episodes (mean)	2	6	4
# Manic episodes (mean)	4	6	5
N Psychosis cases (%)	72 (24.2)	270 (48.6)	342 (40.1)
N Alcohol abuse cases (%)	18 (6.1)	122 (21.9)	140 (16.4)
N Substance abuse cases (%)	30 (10.1)	105 (18.9)	135 (15.8)
N Suicidal ideation cases (%)	75 (25.3)	256 (46.0)	331 (38.8)
GWAS2			
N Effective sample (% from total)	309 (24.6)	949 (75.4)	1258
Females (%)	157 (50.8)	552 (58.2)	709 (56.4)
Age (mean ± SD)	48 ± 15	46 ± 13	47 ± 14
Age-at-onset (mean ± SD)	25 ± 10	25 ± 11	25 ± 11

**Table 2:** Summary findings from the genetic association meta-analyses of Li responses in ConLi^+^Gen.

Meta-analysis summary	Response vs No-response	Continuous Li response
# SNPs after QC	557,037	625,818
# SNPs p < 0.05	27,426	31,489
# SNPs p < 1×10^−4^	124	33
# Lead SNPs	11	11
Top lead SNP	rs4313539	rs12776537
Effect allele	C	A
p-value	2.1×10^−6^	2.5×10^−5^
Z	4.7	−4.2
Gene	*FAT3*	*BMPR1A*
# SNPs p < 1×10^−5^	15	0

**Table 3:** Summary of findings from the association analyses of the ImmuneSet with clinical characteristics in the ConLi^+^Gen “GWAS1” sample.

Phenotype	N	Exploratory threshold (p < 1e−4)		Suggestive GWAS threshold (p < 1e−5)			Top gene	
		# SNPs	# Genes	# SNPs	# Genes	Genes	Symbol	Pval
Age-at-onset	853	54	21	3	2	GRK5, PLD3	GRK5	3.9×10^−6^
# Depressive episodes	692	107	31	54	6	BLNK, PHLPP1, ZCCHC11, SACS, CPPED1, PRPF38A	BLNK	1.7×10^−7^
# Manic episodes	665	116	32	14	4	CNTN6, KALRN, LY86, PTK2B	CNTN6	2.4×10^−6^
Psychosis	692	45	13	1	1	DSCAM	DSCAM	7.6×10^−6^
Alcohol abuse	835	29	9	0	0	-	SCD5	1.8×10^−5^
Substance abuse	832	78	17	15	3	TPD52, NOD1, XKR4	TPD52	4.510^−7^
Suicidal ideation	660	30	7	13	1	JARID2	JARID2	3.9×10^−6^

**Table 4:** Prioritized ImmuneSet candidate genes for Li response and clinical characteristics in ConLi^+^Gen.

Gene	Chr	Start	End	Priority	Phenotypes
*ALK*	2	29415640	30144432	Psychiatric	Depression, Mania
*NRXN1*	2	50145643	51259674	Psychiatric	Depression, Hypomania, Substance
*RTN4*	2	55199325	55339757	Psychiatric	Mania, Psychosis
*CNTNAP5*	2	124025287	124915287	Li response	LiResponse, Alda_B, Alda_Total
*LRP1B*	2	140988992	142889270	Psychiatric	AAO, Mania
*ROBO2*	3	75906695	77649964	Both	Alda_Total, Hypomania, Substance
*BANK1*	4	101411286	102074812	Both	Continuous.LiResp, Alda_A, Alda_B, Alda_Total, Hypomania
*CDH12*	5	21750673	22853622	Both	LiResponse, Suicide
*DSP*	6	7541575	7586717	Li response	Continuous.LiResp, Alda_Total
*NPSR1-AS1*	7	34386124	34911194	Psychiatric	Depression, Suicide
*CNTNAP2*	7	146116002	148420998	Both	LiResponse, Alda_Total, Mania
*NRG1*	8	31496902	32622548	Psychiatric	Mania, Substance
*XKR4*	8	56014949	56454613	Psychiatric	Depression, Mania, Psychosis, Substance
*NFIB*	9	14081843	14398983	Li response	Continuous.LiResp, Alda_B
*NRG3*	10	83635070	84746935	Psychiatric	Alcohol, Psychosis
*BMPR1A*	10	86756601	86932838	Li response	Continuous.LiResp, Alda_A
*GRK5*	10	120967101	121215131	Psychiatric	AAO, Depression
*FAT3*	11	92352096	92896470	Both	LiResponse, Alda_Total, Depression
*PCDH9*	13	66302834	67230445	Both	Alda_B, Mania, Substance
*CPPED1*	16	12756919	12897874	Psychiatric	AAO, Depression
*HAS3*	16	69105564	69118719	Li response	Continuous.LiResp, Alda_A, Alda_Total
